# Targeted rehabilitation to improve outcome after total knee replacement (TRIO): study protocol for a randomised controlled trial

**DOI:** 10.1186/1745-6215-15-44

**Published:** 2014-02-01

**Authors:** A Hamish RW Simpson, David F Hamilton, David J Beard, Karen L Barker, Timothy Wilton, James D Hutchison, Chris Tuck, Andrew Stoddard, Gary J Macfarlane, Gordon D Murray

**Affiliations:** 1 Department of Trauma and Orthopaedics, University of Edinburgh, Edinburgh EH16 4SB, UK; 2 Nuffield Department of Orthopaedics, Rheumatology and Musculoskeletal Sciences, University of Oxford, Oxford OX3 7LD, UK; 3 Nuffield Orthopaedic Centre, Oxford University Hospitals NHS Trust, Oxford OX3 7HE, UK; 4 Department of Orthopaedics, Royal Derby Hospital, Derby DE22 3NE, UK; 5 Department of Orthopaedic Surgery, University of Aberdeen, Aberdeen AB25 2ZD, UK; 6 Edinburgh Clinical Trials Unit, University of Edinburgh, Edinburgh EH4 2XU, UK; 7 Epidemiology Group, School of Medicine and Dentistry, University of Aberdeen, Aberdeen AB25 2ZD, UK; 8 Centre for Population Health Sciences, University of Edinburgh, Edinburgh EH8 9AG, UK

**Keywords:** Total knee replacement, Outcomes, Rehabilitation, Stratified treatment

## Abstract

**Background:**

Approximately 20% of patients are not satisfied with the outcome of total knee replacement, great volumes of which are carried out yearly. Physiotherapy is often provided by the NHS to address dysfunction following knee replacement; however the efficacy of this is unknown. Although clinically it is accepted that therapy is useful, provision of physiotherapy to all patients post-operatively does not enhance outcomes at one year. No study has previously assessed the effect of targeting therapy to individuals struggling to recover in the early post-operative phase.

The aim of the TRIO study is to determine whether stratifying care by targeting physiotherapy to those individuals performing poorly following knee replacement is effective in improving the one year outcomes. We are also investigating whether the structure of the physiotherapy provision itself influences outcomes.

**Methods/Design:**

The study is a multi-centre prospective randomised controlled trial (RCT) of patients undergoing primary total knee replacement, with treatment targeted at those deemed most susceptible to gain from it. Use of the national PROMS programme for pre-operative data collection allows us to screen all patients at initial post-operative clinical review, and recruit only those deemed to be recovering slowly.

We aim to recruit 440 patients through various NHS orthopaedic centres who will undergo six weeks of physiotherapy. The intervention will be either ‘intensive’ involving both hospital and home-based functional exercise rehabilitation, or ‘standard of care’ consisting of home exercises. Patients will be randomised to either group using a web-based system. Both groups will receive pre and post-intervention physiotherapy review. Patients will be followed-up to one year post-operation. The primary outcome measure is the Oxford Knee Score. Secondary outcomes are patient satisfaction, functional ability, pain scores and cost-effectiveness.

**Trial registration:**

Current Controlled Trials ISRCTN23357609. ClinicalTrials.gov NCT01849445.

## Background

Knee osteoarthritis is an extremely common and extremely disabling condition, often ultimately requiring surgical intervention. Total knee replacement (TKR) is an increasingly common procedure with over 75,000 knee replacements performed each year in the UK alone [1]. Projections of future surgical volume suggest further great increases year on year [2]. TKR is highly effective at reducing pain and improving physical function in patients with end stage osteoarthritis. Around 20% of patients however report dissatisfaction with their post-operative outcome, twice the rate following total hip replacement [3-6].

Physiotherapy is a commonly employed intervention to address dysfunction following TKR; though the efficacy of this is unknown. A previous study suggested that post-operative physiotherapy was not effective at improving the patients’ one year outcome, when applied uniformly to all patients after knee replacement [7]. Clinically, patients performing poorly post-operatively are often referred for physiotherapy review; however no study has specifically assessed patients that perform poorly post-operatively. Demonstration of the effectiveness of post-operative physiotherapy management in this ‘at risk’ group would provide support for specific policy to address this substantial population.

A recent Cochrane review examined multidisciplinary rehabilitation programmes following hip and knee joint replacement and concluded that home-based care may be beneficial; but stressed the low quality of the current evidence-base and surmised that further high quality research is needed [8]. Unspecified post-operative physiotherapy applied to a whole cohort of patients who had undergone knee replacement did not find a significant improvement over the control group one year following surgery [7]. However as the majority of patients had a good result, it was not possible to determine if targeted intensive physiotherapy would have helped the patients destined to have a poor outcome.

A previous UK economic analysis of a different physiotherapy treatment than is proposed in this trial showed no significant effects, when such physiotherapy is applied universally to all total knee arthroplasty (TKA) patients [9]. The analyses therefore reverted to cost-minimisation analysis, in which assessment is made on the basis of least costs alone. It is entirely possible that subgroups of patients may be cost-effective to treat when the total population is not [10]. Mitchell *et al*. (2005) found the cost of physiotherapy to be small at only £136.50 per patient (95% CI £113 to £160), as such, only a comparatively small change in patient quality of life is required for the treatment to fall comfortably below the threshold of £30,000 per quality adjusted life year (QALY) implied by NICE [11] as being cost-effective.

### Objectives

The primary objective of TRIO is to evaluate if early targeting of post-operative physiotherapy to patients that initially perform poorly following TKA can improve patient outcome at one year following surgery.

Secondary objectives include assessment of patient function, patient satisfaction and the cost effectiveness of delivering enhanced targeted physiotherapy.

## Methods/Design

### Study design

The study is a multi-centre randomised controlled trial comparing the effect of intensive physiotherapy with current standard of care therapy, targeted at patients considered to be performing poorly at six weeks following TKR.

This study makes use of the national patient Patient Reported Outcome Measures (PROMS) programme, whereby all providers of elective knee replacement are required to collect pre-operative data using the Oxford Knee Score (OKS) and EuroQol 5-Dimension score (EQ-5D). All the centres involved in this project collect routine baseline data using these metrics. This allows us to recruit only those poorly performing patients at the initial *post-surgical* clinical review (six weeks post-operation), and not the entire TKR population. These pre-operation data will be accessed retrospectively for recruited patients at the individual centres.

All patients will be made aware of the study pre-operatively at the recruiting centres. Prior to surgery, they will complete the routine pre-operative outcome assessment questionnaires (OKS and EQ-5D) as part of the national PROMS program and then undergo the local standard TKR and immediate post-operative care pathway. All patients will be routinely reviewed six weeks post-operatively by the usual clinical teams. At this review the OKS will again be assessed. Those patients who report a score of 26 or less (on the 0 to 48 OKS scoring system), which is defined as poor by the modified Kalairajah classification [12], will be approached to consent. If consent is given and they are eligible to enter the trial, they will be randomised to either standard care (defined in this study as: initial physiotherapy review, six weeks of home exercise prescription and final review) or an interventional arm, where 18 sessions of structured physiotherapy will be administered over a six week period. Six of these 18 sessions will be ‘contact sessions’ performed under the supervision of the physiotherapist.

Focus groups run by the Arthritis Research UK Osteoarthritis Clinical Study Group in the preparation phase of this trial identified that the standard care provision of physiotherapy post-arthroplasty varies across the UK and even within local healthcare authorities. We therefore standardised the control arm to be an unsupervised home exercise intervention with initial and final physiotherapist review, which the focus group agreed represented a frequent provision across the UK. This approach allows us to comment on the efficacy of ‘contact’ interventional physiotherapy in addition to simple review and exercise provision and to assess the cost-effectiveness of differing ways of providing post-operative physiotherapy to this targeted patient group.

**Figure 1:**
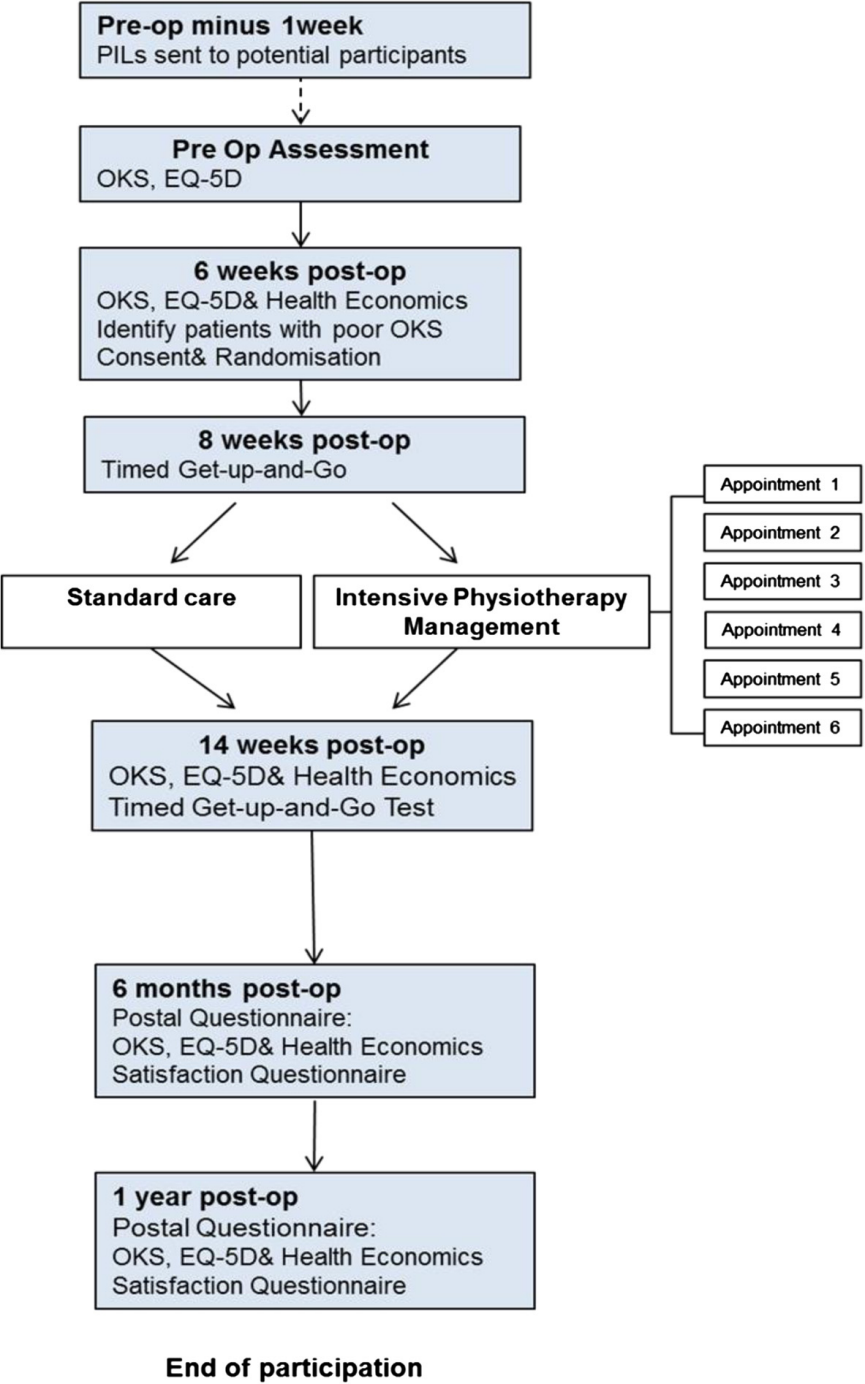
Trial flowchart.

All trial participants will be reviewed immediately post physiotherapy intervention (that is, at 14 weeks post-operation) and then by postal questionnaire at six months and one year post-operation (Figure 1). All post-operative data collection time points are determined by the date of surgery (not the date of trial randomisation). Use of the national PROMS program outcome assessment tools, in addition to their direct applicability, will allow direct comparison of outcome in our interventional group with the wider TKA population.

### Primary outcome measure

The OKS was selected as the primary outcome assessment as it has been developed specifically to measure the outcomes of knee replacement [13,14]. The OKS is a patient reported outcome measure that was designed to measure the impact of pain and functional disability on an individual’s life [15,16]. As such, it is an extensively validated and widely adopted outcome measure in patients undergoing knee replacement surgery and is sensitive to detect changes over time. The OKS is also the most appropriate tool as it is the chosen PROM for this population in the National Joint Registry.

### Secondary outcome measures

Global knee pain severity will be assessed using an 11 point (0 to 10) Visual Analogue Scale (VAS), where 0 represents no pain and 10 the worst possible pain. The validity and sensitivity of the VAS has been well documented [17]. It has been suggested that using multiple measurements of pain status as opposed to a single value of ‘current pain’ may provide more realistic and meaningful measurements of pain intensity [18]. Separate assessments are made of ‘worst pain’ and ‘perceived mean daily pain’ as has been specifically recommended for use in osteoarthritis (OA) clinical trials [19].

The Timed-get-up-and-go test (TUG) is a simple test used to assess a person's mobility, requiring both static and dynamic balance. It is the time that a person takes to rise from a chair, walk three meters, turn around, walk back to the chair, and sit down. TUG performance has been found to decrease significantly with mobility impairments, while residential status and physical mobility status have been determined to be significant predictors of TUG performance [20]. The TUG has excellent inter-rater and intra-rater reliability [21]. The test score also correlates well with gait speed and scores on balance assessments.

Patient satisfaction will be assessed with a specific patient satisfaction question. The overall satisfaction response is thought to be primarily influenced by patient pain, function and meeting of pre-operative expectations [3], thus a further four additional specific sub-questions relating to these facets of satisfaction will also be asked to explore the effect of the trial intervention.

Health economic analysis will compare the cost-effectiveness over one year of providing additional physiotherapy, as described above, against standard care for those patients defined as performing poorly by their Oxford Knee Score after six weeks. A cost-utility analysis (calculation of incremental costs per quality adjusted life years (QALY) gained) will be performed from the NHS perspective in line with the preferred method by NICE [22]. The EQ-5D questionnaire [23] and bespoke health economic questionnaire will be used to collect these data. Resource use estimates from the study will be combined with unit costs taken from standard costing sources to produce an estimate of the cost to the NHS of providing the treatment.

### Study population

Four hundred and forty patients identified at six week post-operative clinical review and defined as performing poorly will be recruited to the study. The planned recruitment period is 18 months.

### Inclusion criteria

1. Patients undergoing primary TKA for osteoarthritis.

2. Defined poor outcome (OKS less than or equal to 26) at first post-operative review (six weeks).

3. Patients are able to consent and willing to comply with the study protocol.

### Exclusion criteria

1. Patients undergoing revision knee arthroplasty or fully constrained knee arthroplasty.

2. TKR for a diagnosis other than osteoarthritis.

3. Patients unable to attend the study physiotherapy intervention centres.

4. Procedures done purely for pain relief (such as for patients with no walking capacity).

5. Patient’s already receiving on-going structured post-operative exercise rehabilitation.

### Recruitment and consent

Patients will be made aware of the trial at time of surgical pre-admission. All patients attending six week post-surgical review clinic will be screened for eligibility, and if suitable, invited to participate in the trial. Written informed consent will be obtained by a suitably qualified member of the research team at study entry.

### Randomisation procedures

Randomisation will be carried out using a bespoke web-based randomisation protocol. Patients will be randomised on a 1:1 ratio to either ‘standard of care physiotherapy’ or ‘enhanced physiotherapy’. Randomisation will be stratified by centre with block allocation.

### Intervention

The specific rehabilitation protocol we will employ for TRIO is based on the current best evidence of functional rehabilitation [24]. This incorporates four categories: (1) range of motion, (2) strengthening (3) proprioception and (4) balance/gait. The specific exercises the therapist will use to achieve defined goals for each category are outlined in detail in Table 1.

**Table 1:** Rehabilitation protocol

**Category**	**Description**	**Expected achievements**
Range of motion	• Prone knee flexion AROM	• AROM of 100
	• Heel props for extension PROM	• Less than 1cm effusion after exercises
	• Stationary bicycle/rowing machine for ROM stimulus and endurance	
		• Able to attain full extension
	• Hamstring, quadriceps & gastrocnemius /soleus stretching	
	• Total end range time (TERT) of 30 minutes a day until ROM guideline attained (Aim 100°)	
Strengthening	• Isometric quads	• Able to achieve voluntary quadriceps control, demonstrated by SLR without lag
	• Straight leg raise (if not able)	• Able to perform semi squat equal weight bearing between limbs
	• Partial squats to 90°	
	• Supine sub-maximal leg press or equivalent (emphasis on pain free motion and neuromuscular control vs. pure strengthening)	
	• Front and lateral step ups progressing from 10cm	
	• Resistive exercises against Theraband 90°-30° in sitting – progress to 90°-0°	
Proprioception	• Balance exercises in single leg stance	• Able to perform sit-to-stand unsupported
	• Sit to stand	• Able to perform single leg stance activities
	• Balance ball	
	• BalanceMaster / low wobbleboard if BalanceMaster not available	
	• Perturbation from soft unstable surface	
Balance/Gait	• Braiding – alternate front and back crossover steps whilst moving laterally – progress by increasing speed	• Able to complete multiple changes of direction walking without support
	• Tandem Walk forward and backwards	
	• Walk multiple change in direction on command	
	• Shuttle walking to increase stamina	

Patients in the interventional arm of the trial will see their physiotherapist once a week for structured rehabilitation; they will be expected to undertake a further two sessions of specified exercise according to the trial exercise prescription. This additional rehabilitation will be directed by the physiotherapist and reviewed at the weekly ‘contact’ session. This protocol is in marked contrast with the ‘routine standard of care’ arm, which consists of home-based exercises involving bending the knee in isolation and using the weight of the limb to strengthen the quadriceps muscle with a static knee. The participant will be asked to perform 18 sessions of unsupervised home exercise in this trial arm. For the stated ‘non-contact’ physiotherapy sessions (in both arms of the trial), the patient will be given a rehabilitation diary to document the exercise they undertake. Extra or additional physiotherapy will not be offered to the control group within the trial period. The activities of both groups will be recorded, and any patients in the control arm receiving extra physiotherapy will be documented.

### Study assessments

All the study centres involved in this project collect routine baseline data using the OKS and EQ-5D. These data will be accessed retrospectively for recruited patients at the individual centres.

At the study baseline (six weeks post-operation) an OKS will be completed and eligible patients consented and randomised. Once consent has been obtained, the additional trial Health Economics Questionnaire and EQ-5D questionnaire will be completed. Physiotherapy pre-intervention assessment will occur two weeks following this (and TUG test performed). Following either study intervention, all patients will have a second (post-intervention) physiotherapy review (OKS, TUG, EQ-5D and Health Economics Questionnaire). At six months and one year post-operation, the OKS, EQ-5D, Health Economics and Satisfaction questions will be assessed by postal questionnaire (Table 2).

**Table 2:** Study assessments by time point

**Time point**	**Pre-operation**	**6 weeks**	**8 weeks**	**14 weeks**	**6 months**	**1 year**
OKS	X	X		X	X	X
EQ-5D	X	X		X	X	X
Patient demographics		X				
Pain score VAS		X		X	X	X
Health economics questionnaire		X		X	X	X
TUG			X	X		
Satisfaction questionnaire					X	X

Trial baseline is at six weeks post-operation. Pre-operative data will be accessed retrospectively. Assessment timeframes are calculated from the date of surgery.

### Safety assessment

As the study is a non-Clinical Trial of an Investigational Medicinal Product (CTIMP) with an intervention of additional physiotherapy, there is not deemed to be a significant risk to patient safety. In the case of adverse events, participants will be advised to seek medical advice through their general practitioner. Specific adverse events such as treatment withdrawals and missed treatments due to pain will be collected through the study case report form. Patient reported outcomes will measure knee pain using a Visual Analogue Scale (VAS) and global health state via the EQ-5D.

### Sample size calculation

The sample size for TRIO is 400 patients (200 per arm). This was determined from previous research. The power calculation (Table 3) is based on the accepted change in the OKS that is considered clinically significant and of interest when comparing interventions (3 points) [16]. Typical one year change scores for the OKS are 16 points with a reported standard deviation of 9.2 [3,4].

**Table 3:** Power calculation

**Difference**	**Size**	**Power**
3	150	80%
3	200	90%

Two-sample *t*-test, alpha = 0.05 (two-sided), assumed SD = 9.2.

A large prospective cohort is required to achieve the desired numbers of poorly functioning knees; therefore the study will recruit from multiple major centres. Based on reported poor outcome rates of approximately 20%, and allowing for a potential of up to 20% of eligible patients being unwilling to be randomised, we aim to screen 2,750 patients prospectively at the six-week post-surgical review to identify 550 eligible, poorly functioning patients and to achieve recruitment (randomisation) of 440. Allowing for a further 10% loss to follow-up in the RCT will yield the 400 patients we require.

### Statistical analysis

The difference in mean OKS between the groups at one year will be estimated using analysis of covariance (ANCOVA) to adjust for baseline OKS at randomisation (that is, at six weeks). The results will be presented as an adjusted mean difference with its corresponding 95% confidence interval. The principal analysis will be on an intention to treat basis where participants will be analysed according to the allocated group using all available data. Additional summary statistics will also be presented for change in OKS from pre-operative values.

Health economic analysis will compare the cost-effectiveness (over one year) of providing targeted additional physiotherapy to those patients defined as performing poorly after six weeks against standard care. A cost-utility analysis (calculation of incremental costs per quality adjusted life year (QALY) gained) will be performed from the NHS perspective in line with the preferred method by NICE [22]. Resource use estimates from the study will be combined with unit costs taken from standard costing sources to produce an estimate of the cost to the NHS of providing the treatment. Health benefits in terms of QALYs will be derived from the standard UK tariff for the EQ-5D [25]. Estimates of NHS cost and QALYs will then be combined to produce an incremental cost-effectiveness ratio. This ratio will be presented using probabilistic sensitivity analysis [26] with deterministic analysis used on key modelling assumptions.

### Ethics

TRIO has received ethical approval from the National Research Ethics Service, South East Scotland Research Ethics Committee 01 in April 2013 (REC reference: 13/SS/0051).

## Trial status

Recruitment to TRIO commenced in September 2013 and is on-going at the time of manuscript submission. The expected time of recruitment completion is March 2015.

## Abbreviations

ANCOVA: analysis of covariance; CTIMP: Clinical Trial of an Investigational Medicinal Product; EQ-5D: EuroQol 5 Dimension score; NICE: National Institute for health and Care Excellence; OA: osteoarthritis; OKS: Oxford Knee Score; PROMS: Patient Reported Outcome Measures; QALYs: Quality Adjusted Life Years; RCT: randomised controlled trial; TKA: total knee arthroplasty; TKR: knee replacement; TUG: Timed-get-up-and-go test; VAS: Visual Analogue Scale.

## Competing interests

The authors declare that they have no competing interests.

## Authors’ contributions

HS is the chief Investigator, DH, DB, KB, TW, JH, GMF and GM were co-applicants on the grant application to ARUK, CT is the trial manager, AS is the trial health economist. All authors were involved in the study design and implementation. HS and DH were responsible for writing this manuscript; all authors reviewed and approved the final manuscript.
